# Preparation, Characterization and Photocatalytic Activity of La-Doped Zinc Oxide Nanoparticles

**DOI:** 10.3390/ma12081195

**Published:** 2019-04-12

**Authors:** Loan T. T. Nguyen, Lan T. H. Nguyen, Anh T. T. Duong, Bui Duc Nguyen, Nguyen Quang Hai, Viet Ha Chu, Trinh Duy Nguyen, Long Giang Bach

**Affiliations:** 1 Faculty of Chemistry, Thai Nguyen University of Education, Thai Nguyen City 24000, Vietnam; nguyenhienlan@dhsptn.edu.vn (L.T.H.N.); duongtuanh@dhsptn.edu.vn (A.T.T.D.); ducnguyen@dhsptn.edu.vn (B.D.N.); 2 Faculty of Physics, Thai Nguyen University of Education, Thai Nguyen City 24000, Vietnam; nguyenquanghai@dhsptn.edu.vn (N.Q.H.); chuvietha@tnu.edu.vn (V.H.C.); 3 NTT Hi-Tech Institute, Nguyen Tat Thanh University, Ho Chi Minh City 70000, Vietnam; blgiang@ntt.edu.vn; 4 Center of Excellence for Green Energy and Environmental Nanomaterials, Nguyen Tat Thanh University, Ho Chi Minh City 70000, Vietnam; 5 Center of Excellence for Functional Polymers and NanoEngineering, Nguyen Tat Thanh University, Ho Chi Minh City 70000, Vietnam

**Keywords:** La-doped zinc oxide, nanoparticles, gel combustion, optical property, photocatalytic activity

## Abstract

Lanthanum (La)-doped zinc oxide nanoparticles were synthesized with different La concentrations by employing a gel combustion method using poly(vinyl alcohol) (PVA). The as-synthesized photocatalysts were characterized using various techniques, including X-ray diffraction (XRD), transmission electron microscopy (TEM), energy dispersive X-ray analysis (EDX), photoluminescence (PL) spectroscopy, and UV–visible absorption spectroscopy. The average size of ZnO nanoparticles decreased from 34.3 to 10.3 nm with increasing concentrations of La, and the band gap, as evaluated by linear fitting, decreased from 3.10 to 2.78 eV. Additionally, it was found that the photocatalytic activity of doped samples, as investigated by using methyl orange dye under visible lights, improved in response to the increase in La concentration. The decomposition of methyl orange reached 85.86% after 150 min in visible light using La0.1Zn0.9O as the photocatalyst.

## 1. Introduction

Among n-type semiconductors, zinc oxide is known for its advantageous characteristics, including a wide direct band gap (3.27 eV), large exciton binding energy (about 60 meV), high optical gain at room temperature, great saturation velocity, piezoelectricity, and pyroelectricity. Zinc oxide is also non-toxic, biocompatible, and simple and inexpensive to synthesize [1]. These properties of zinc oxide make it an important II-VI semiconductor. Due to a myriad of versatile applications in technical manufacturing, cosmetics, and the pharmaceutical industry, zinc oxide nanomaterials, such as nanoparticles or zinc oxide-based semiconductors, have been extensively studied [2,3].

Zinc oxide nanomaterials have been synthesized by different techniques such as the co-precipitation method [4,5], sol–gel method [6,7,8], combustion method [9], hydrothermal method [10], and thermal decomposition [1], depending on the desired structure. ZnO, when modified, could be utilized as a base material for diluted magnetic semiconductors [11], gas sensors [12,13], and photocatalysts [14,15,16,17]. In photocatalytic applications, ZnO nanoparticles were used to remove numerous azo dyes, such as methylene blue [18], methyl orange [17], rhodamine B [19], and methyl red [20].

The main drawback of ZnO, when used as a photocatalyst, is the absorption wavelength of the material, which is limited in the ultraviolet region. This induces excessive electron–hole recombination. In addition, ZnO could also promote photochemical corrosion [21]. 

Depending on structural properties and chemical composition, the photocatalytic properties of ZnO may vary with particle size, morphology, and crystallinity of the materials. Modifications such as thermal treatment could result in changes, or improve the photocatalytic activities in some cases. Another possibility is to dope the materials with rare earth ions to modulate the properties of ZnO. This could enhance photocatalytic activity through limiting the recombination of electron–hole pairs and improving the absorption of light [22].

Previous studies claim that the photocatalytic characteristics of doped ZnO can be enriched with a moderate amount of rare earth elements such as La [23,24,25], Eu [22], Ce [26], and Sm [27]. Among them, La-doped ZnO materials are distinguished by their gas sensitivity and photocatalytic activity, as evidenced by studies in which various synthesis routes have been adopted [28,29,30]. Specifically, Jia et al. [28] solvothermally synthesized La^3+^-doped ZnO materials and examined the photocatalytic activities of the product against rhodamine B (RhB) with respect to different doping concentrations. The results showed that doped materials had substantially enhanced photocatalytic efficiency and that 2% La was the optimal amount of doping required to achieve peak RhB degradation. In another study [29], a combination of electrospinning and the calcination method was adopted to produce ZnO nanomaterials incorporated with La^3+^ ions, which showed higher photocatalytic activity in comparison with that of undoped ZnO. In addition, the optimal removal level of Congo red dye was found to be 97.63%, achieved at a 2% concentration of La^3+^ doping, with a catalyst dosage of 0.283 g/L and with an initial dye concentration of 10.5 mg/L. These results are confirmed by a study using similar methods, where the use of La, Er, and Sm as dopants, at 1% concentration, was compared to the use of ZnO [30]. At this doping concentration, Sm was shown to be the superior dopant, demonstrated by the higher photodegradation (95.8%) of Congo red. However, this figure is still lower than the photodegradation performance delivered by La-doped ZnO at 2%.

La doping of ZnO is also suggested for other applications regarding food sanitization, medicine, and wastewater treatment. In a study of Bomila et al. [31], where La-doped ZnO nanoparticles were tested for antibacterial activity, the doping of La was found to significantly improve the antibacterial activity of the materials against *Salmonella typhi* and *Bacillus subtilis* bacteria in comparison to the bare ZnO. Furthermore, it was also revealed that the antibacterial activity improves with decreasing particle size due to the active species produced by photoreactions. In another study, 3% La-doped ZnO nanoparticles also elicited relatively high cytotoxicity against three cancer cell lines (MDA-MB-231, KCL-22, and HeLa cell lines) [32].

In this report, we aim to investigate the structure and the optical and photocatalytic properties of ZnO materials, and compare them with those obtained from ZnO materials doped with La. In line with existing studies, the materials were synthesized by a gel combustion method using poly(vinyl alcohol) (PVA) as a chelating agent and as a fuel. 

## 2. Results and Discussion

### 2.1. X-ray Diffraction (XRD) Study

**Figure 1:**
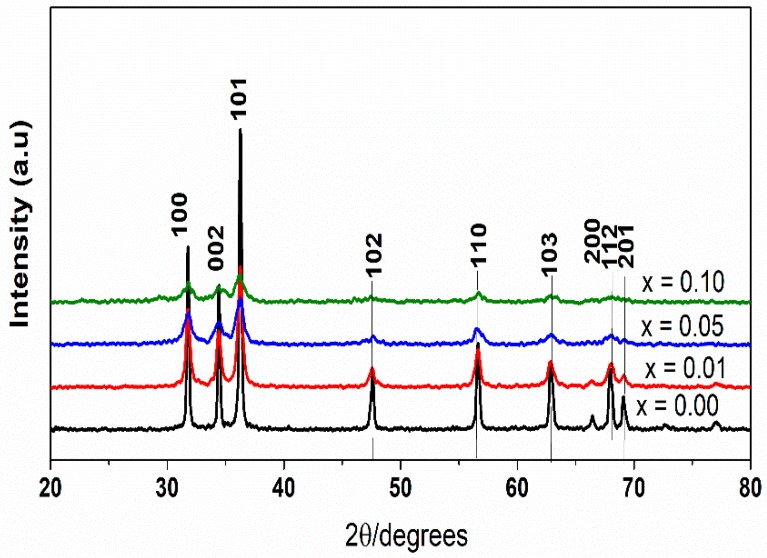
X-ray diffraction (XRD) patterns of the La_x_Zn_1−x_O samples calcined at 500 °C.

Figure 1 shows the powder XRD spectra of La_x_Zn_1−x_O (x = 0.00, 0.01, 0.05, 0.10) nanoparticles with a 2θ angle ranging from 20° to 80°. Numerous diffraction peaks found at 2θ = 31.86° (100), 34.54° (002), 36.32° (101), 47.62° (102), 56.66° (110), 62.96° (103), 66.44° (200), 68.04° (112), 69.18° (201), according to JCPDS card No. 36-1451, could be assigned to the hexagonal wurtzite structure of ZnO [28,33,34]. In addition, the XRD spectra indicated the absence of lanthanum oxide or Zn–La alloys formation [35], and also indicated a trend of continuously broadening XRD peaks of La-doped ZnO in response to increasing La load. Therefore, it is suggested that the dopant was dispersed uniformly in the ZnO matrix, causing no alteration to the crystal structure [25,36]. Table 1 compares pure ZnO and La-doped ZnO with different dopant concentrations, in terms of crystallite sizes and lattice parameter values. It is interesting to note that the crystallite sizes and lattice parameter values of La-doped ZnO, as resulting from the Scherrer equation, were lower than those of pure ZnO, which is inversely proportional to the La doping. This was thereby consistent with previous studies [23,25,28]. The reduction of particle size and lattice parameters of La-doped ZnO could be attributable to the diffusion and growth impairment of ZnO crystal grains, which is caused by the the ionic radius 0.74 and 1.26 Å, respectively, for Zn^2+^ and La^3+^ [23,28]. These size characteristics make La-doped ZnO materials beneficial regarding photocatalytic activity.

### 2.2. Energy Dispersive X-ray Analysis (EDX) Study

**Figure 2:**
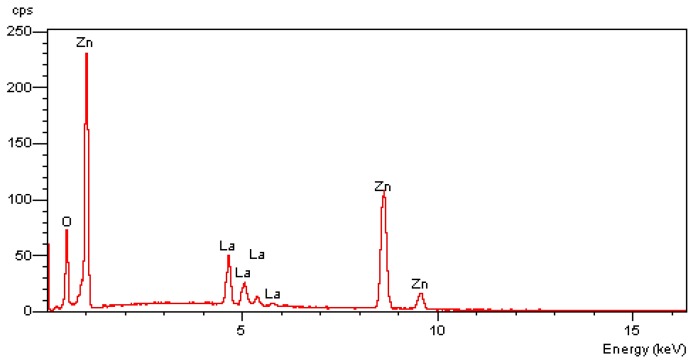
Energy dispersive X-ray (EDX) spectrum of La0.05Zn0.95O nanoparticles.

EDX results showing the chemical purities and elemental composition of the La_0.05_Zn_0.95_O materials are displayed in Figure 2. The EDX spectra of the nanoparticles indicated the existence of Zn, O, and La elements, as confirmed by their corresponding peaks and the absence of other characteristic peaks. The produced sample was also devoid of other element impurities, due to the EDX data showing that there was 38.65% and 4.8% of zinc and lanthanum, respectively. 

### 2.3. Transmission Electron Microscopy (TEM) Study

**Figure 3:**
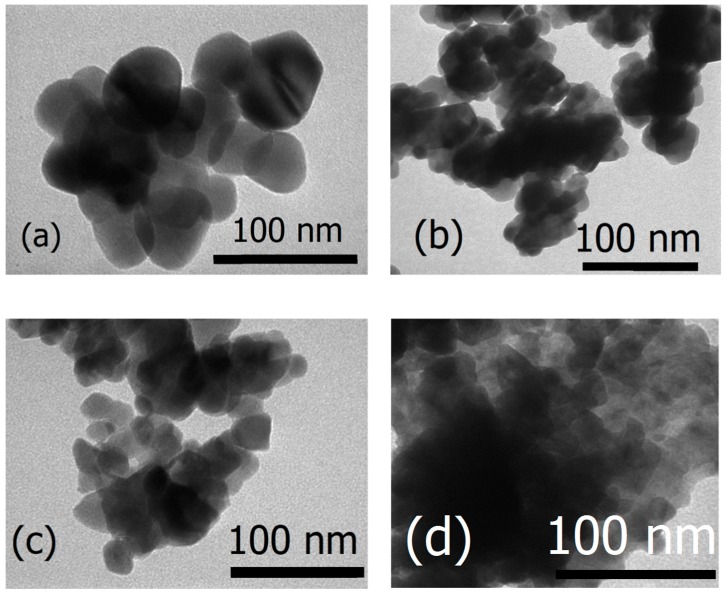
Transmission electron microscopy (TEM) images of the La_x_Zn_1−x_O samples: x = 0.0 (**a**); x = 0.01 (**b**), x = 0.05 (**c**), and x = 0.1 (**d**).

Figure 3 shows Transmission Electron Microscopy (TEM) images of the pure and La-doped ZnO samples. The drastic difference between the TEM images of samples synthesized with varying La concentrations suggests that intensifying the La doping on ZnO reduces the size of ZnO nanoparticles and induces no change in morphology. This reduction in crystalline size was also supported by the XRD results.

### 2.4. UV–Visible Absorption Study

**Figure 4:**
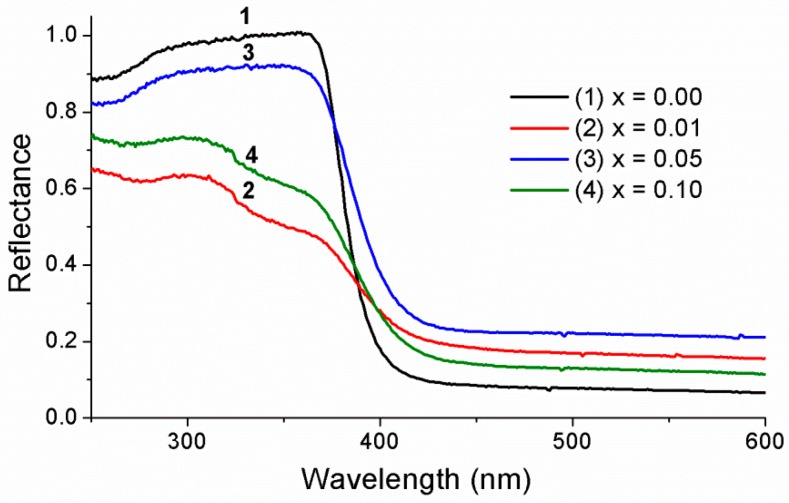
The UV–vis diffuse reflectance spectra of La_x_Zn_1−x_O nanoparticles.

The optical properties of pure ZnO and ZnO doped with different La concentrations were explored using UV–visible absorption spectroscopy. Figure 4 shows that the absorbance in the ultraviolet region (200–400 nm) was high for both pure ZnO and La-doped ZnO. In the visible region (400–800 nm), the absorbance of La-doped ZnO samples was improved. From the spectra, the band gap of La_x_Zn_1−x_O samples were determined from the Kubelka–Munk equation via conversion to absorbance [3]. For the synthesized catalyst, the band gap energy E_g_ (eV) was calculated using the following equation.

(1) $$E_{g}=\frac{h.c}{\lambda_{\max}}=\frac{1240}{\lambda_{\max}},$$

where h, c, and λ_max_ are the Planck constant $\left(6.62.10^{-34}\;J.s.\mathrm{photon}{}^{-1}\right)$, speed of light (3 × 10^8^ m.s^−1^), and wavelength at the absorption edge (nm), respectively [37]. 

Table 2 shows the band gap results of both pure ZnO and La-doped ZnO. Evidently, the band gap of La-doped ZnO decreased gradually from 3.1 to 2.78 eV while La concentration increased from 0 mol % to 10 mol %. This is in line with the results of La-doped TiO_2_ in a previous study [38]. Regardless of the higher radius of La^3+^ in comparison with that of Zn^2+^, La^3+^ could easily enter ZnO lattices due to the low decomposition temperature of nitrate (126 °C), which facilitates the movement of the formed La_2_O_3_ into ZnO lattices during the annealing process. La^3+^ ions that enter the lattice could either take over the Zn^2+^ position or be situated in the interstitial voids [39]. 

### 2.5. Photoluminescence Spectrum Analysis

**Figure 5:**
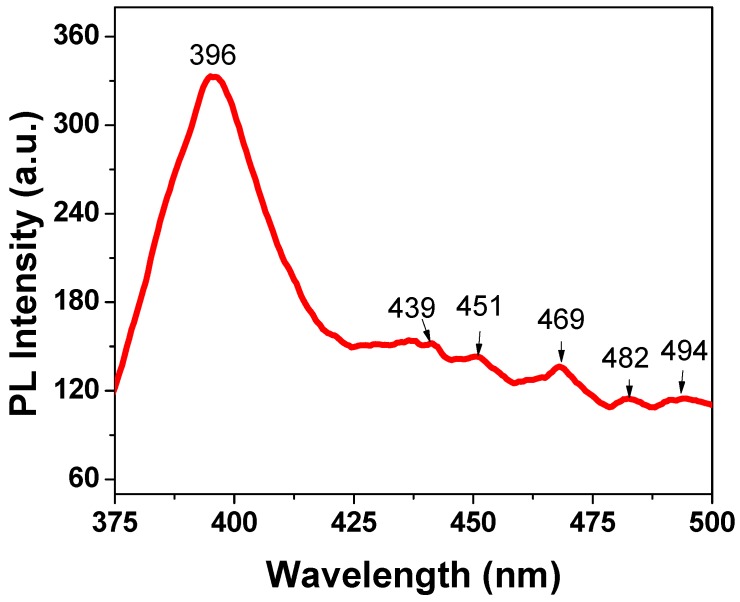
Photoluminescence (PL) spectrum of La_0.01_Zn_0.99_O nanoparticles.

The measurements of photoluminescence (PL) spectra of La-doped ZnO nanoparticles were carried out under excitation of 325 nm at room temperature. Figure 5 presents the PL spectrum of 1% La-doped ZnO sample. The PL spectrum showed an emission maximum at 369 nm with very strong intensity, which is attributed to the emission of pure ZnO. When La^3+^ ions were doped into ZnO crystal lattices, interstitial zinc ions and oxygen vacancies were formed along with the incorporation of La^3+^ [40]. The emission peaks at 439, 451, and 469 nm are attributed to the transition between the vacancies of oxygen and interstitial oxygen [28,40]. The peaks at 482 and 494 nm may have originated from the ionized oxygen vacancies in the valance. The results show that the doping of La into ZnO nanoparticles was successful.

### 2.6. Photocatalytic Activity of La-Doped ZnO Nanoparticles

**Figure 6:**
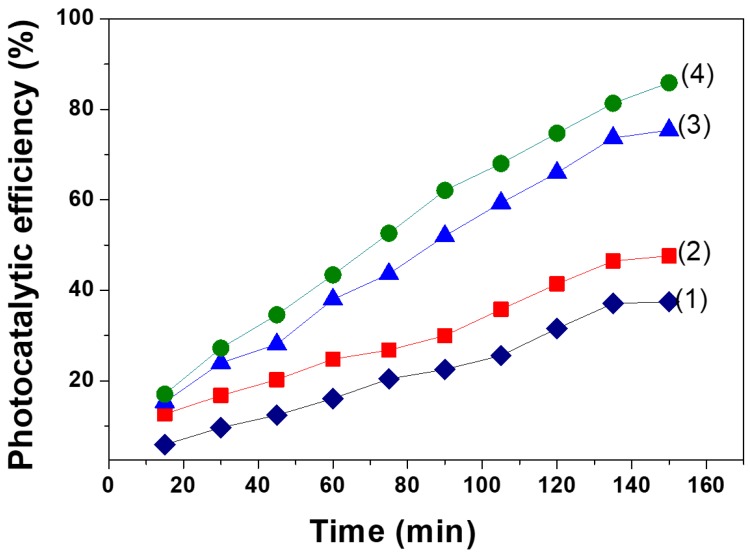
Photocatalytic efficiency of methyl orange (MO) versus time (min) under visible light irradiation on the La_x_Zn_1−x_O nanoparticles: ZnO (1); La_0.01_Zn_0.99_O (2); La_0.05_Zn_0.95_O (3); La_0.10_Zn_0.90_O (4).

Comparison of the photocatalytic efficiency of methyl orange (MO) between pure ZnO and La-doped ZnO nanoparticles was performed in the presence of visible light at different irradiating intervals. The results shown in Figure 6 suggest an improved photocatalytic efficiency of MO in response to increasing irradiation time and La concentration. Higher photocatalytic activities of the La-doped ZnO photocatalysts, compared to that of pure ZnO, were observed, confirming the enhancing effect of La doping on the photocatalytic activity of ZnO photocatalysts. This effect was also observed by L. Elsellami et al. in the case of the 4-nitrophenol degradation in the presence of the La-doped TiO_2_ and visible light [38].

The photocatalytic efficiency of MO reached 85.86% after 150 min under irradiation of visible light using La_0.1_Zn_0.9_O as a photocatalyst. Table 1 indicated that the average crystallite size and lattice parameter values of La_0.1_Zn_0.9_O were smaller than that of the other samples. In addition, Figure 2 indicated that the band gap energy of La_0.1_Zn_0.9_O at 2.78 eV was smaller than that of the other samples. This suggests that both particle size and the specific surface areas of ZnO nanoparticles could be efficiently enhanced by La doping. The doping could increase the number of electrons/holes escaping to the surface of the La_0.1_Zn_0.9_O nanoparticles and, consequently, improve the photodegradation efficiency of La_0.1_Zn_0.9_O.

The rate of reactions could be described as follows:

(2) $$\frac{\mathrm{dC}}{\mathrm{dt}}=kC_{t},$$

where C_t_ and k is the concentration of MO and rate constant of the reaction, respectively. The subscript t denotes time interval. By integration, the equation could be transformed as follows:

(3) $$\ln\frac{C_{o}}{C_{t}}=\mathrm{kt}.$$

**Figure 7:**
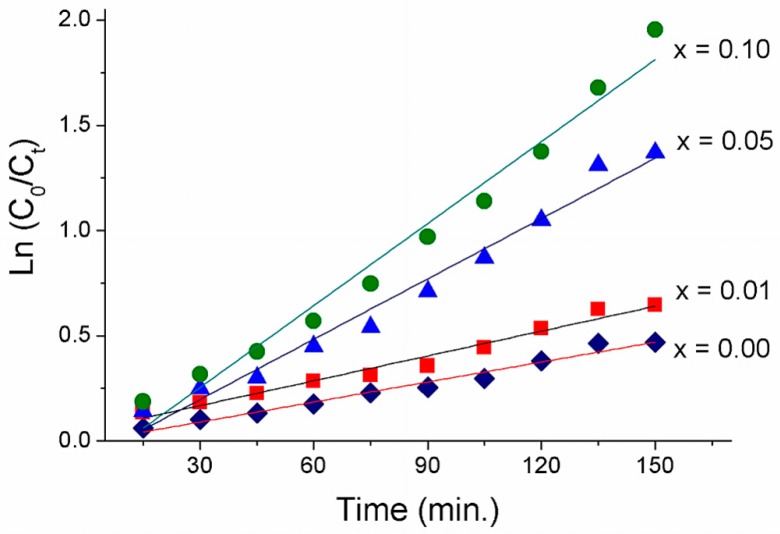
ln(Co/Ct) versus time (min) of MO degradation under visible light irradiation on the La_x_Zn_1−x_O nanoparticles.

Figure 7 plots the variations of ln(C_o_/C_t_) with respect to irradiation time (t) for all the samples. Visually, the relationship could be observed to assume a linear function, which suggests that a pseudo-first-order reaction is suitable for describing the photodegradation of MO [37,41]. The estimation parameters, including k values and the coefficient of determination (R^2^), are shown in Table 3. The increase in the La concentration, from 1.0 mol % to 10 mol %, resulted in a gradual increase in rate constant, from 0.0039 to 0.013 min^−1^. Peak photocatalytic activity was observed at 10 mol % La-doped ZnO, with a rate constant four times larger than that of pure ZnO. Thus, the cause for the improved photocatalytic performance could be two-fold. First, it could be attributable to the combinational effect of La and ZnO. Second, the decreased size and band gap energy of nanoparticles could suppress the recombination rate of the electron–hole pairs. 

## 3. Materials and Methods 

All chemicals, including zinc nitrate tetrahydrate (Zn(NO_3_)_2_.4H_2_O), lanthanum nitrate hexahydrate (La(NO_3_)_3_.6H_2_O), poly(vinyl alcohol) (PVA, MW = 98000 g·mol^−1^), and methyl orange (C_14_H_14_N_3_NaO_3_S) were obtained from Merck (Kenilworth, NJ, USA) and used as received, without further purification.

### 3.1. Preparation of La-Doped ZnO Nanoparticles

A gel combustion method was employed to produce the La-doped ZnO nanoparticles. A typical experimental procedure is described as follows: After PVA was dissolved in deionized water, a pre-specified amount of Zn(NO_3_)_2_.4H_2_O, La(NO_3_)_3_.6H_2_O, according to mol ratio correlative, was added into the above solution with vigorous stirring to form a mixed solution. Afterwards, the mixed solution underwent stirring for 4 hours to form the gel, which was subsequently subjected to thermal treatment at 80 °C for 10 hours in an oven. The produced white precipitate was calcined at 500 °C for 3 hours with a heating rate of 5 °C /min [35].

### 3.2. Characterizations

A D8 Advance diffractometer (Brucker, Madison, WI, USA) was used to produce XRD spectra, characterizing the phase of the obtained powder with Cu Kα radiation (λ = 1.5406 Å) in a 2θ angle ranging from 20° to 80° with a step of 0.03°. The crystallite size, D of ZnO, was determined using the Scherrer formula, in which λ is the X-ray wavelength (0.1504 nm), k is Scherrer’s constant (k = 0.89), β is the full width at half maximum observed in radians, and θ is the angle of diffraction of the (101) peak with the highest intensity. 

Transmission electron microscopy (JEOL-JEM-1010, Tokyo, Japan), UV–vis absorption spectroscopy (U-4100, Hitachi, Tokyo, Japan), photoluminescence (PL) spectroscopy (FL32, Horiba, Kyoto, Japan), and energy dispersive X-ray spectroscopy (JEOL JED 2300 Analysis Station, Tokyo, Japan) were employed to characterize the morphology (TEM and UV–vis,) optical absorption, and the elemental composition of the samples, respectively.

### 3.3. Photocatalytic Degradation of Methyl Orange

The conditions in which the photocatalytic experiments were conducted included continuous circulation mode, ambient temperature, and at the natural pH of methyl orange (MO) solution (pH = 4). Prior to the photocatalytic reaction, the suspension underwent stirring for 45 min in the dark condition to achieve adsorption/desorption equilibrium. Following that, 50 mg of La_x_Zn_1−x_O was dispersed in 100 mL of MO aqueous solution (10 mg·L^−1^), and the suspension was exposed to visible irradiation in 150 min using a 20 W Compact Lamp (Philips) with 130 cd. The concentration of MO was determined at 464 nm using an ultraviolet–visible spectrophotometer (UV-1700 Shimadzu, Kyoto, Japan). To determine the photocatalytic efficiency of MO (H), the following equation was adopted.

(4) $$H=\frac{C_{o}-C_{t}}{C_{o}}\times 100\%,$$

where C_o_ is the equilibrium concentration of MO (mg·L^−1^) measured following dark adsorption and C_t_ is the concentration of MO after irradiation for time t.

## 4. Conclusions

Pure ZnO and La_x_Zn_1−x_O (x = 0.01, 0.05, 0.10) were synthesized via the gel combustion method. The amount of lanthanum significantly affected the morphology, optical properties, and photocatalytic activity of ZnO nanoparticles. Transmission electron micrographs showed that the synthesized samples had spherical morphology and that particle size was inversely proportional to La molar percentage.

The doping of lanthanum into the ZnO lattice induced a decline in the band gap of ZnO, from 3.1 to 2.78 eV. These results suggest that the La-doped ZnO nanoparticles could be promising for optic applications extending into the visible wavelength range. The La_0.1_Zn_0.9_O nanoparticles had the highest photocatalytic degradation of MO (85.86%) after 150 min of visible light irradiation. The kinetics of MO photodegradation were consistent with being a first-order reaction.

## Figures and Tables

**Table 1:** Average crystallite size and lattice parameter values of ZnO and La-doped ZnO calcined at 500 °C.

Sample	2θ (degree)	d101 (Å)	FWHM (deg.)	Average Crystallite Size (nm)	Lattice Parameter (a) (Å)	Lattice Parameter (c) (Å)
Pure ZnO	36.268	2.474	0.244	34.3	3.2505	5.216
La_0.01_Zn_0.99_O	36.261	2.475	0.439	18.9	3.2490	5.208
La_0.05_Zn_0.95_O	36.320	2.474	0.695	12.1	3.2447	5.202
La_0.10_Zn_0.90_O	36.260	2.480	0.814	10.3	3.2447	5.214

**Table 2:** Wavelength and energy of the band gap of pure ZnO and La-doped ZnO nanoparticles.

Samples	ZnO	La_0.01_Zn_0.99_O	La_0.05_Zn_0.95_O	La_0.1_Zn_0.9_O
λ (nm)	400	425	440	445
E_g_ (eV)	3.10	2.91	2.82	2.78

**Table 3:** Photocatalytic reaction parameter of pure ZnO and La-doped ZnO nanoparticles.

Sample	Rate Constant (k), min^−1^	R^2^
Pure ZnO	0.0032	0.9792
La_0.01_Zn_0.99_O	0.0039	0.9770
La_0.05_Zn_0.95_O	0.0096	0.9776
La_0.10_Zn_0.90_O	0.0130	0.9771
